# Radiotherapy is associated with improved survival in stage I–II gastric MALT lymphoma after failed or inadequate *Helicobacter pylori* eradication: a single-center retrospective study

**DOI:** 10.3389/fonc.2025.1688877

**Published:** 2026-01-02

**Authors:** Miao Li, Wenjie Wang, Chi Lu, Yujing Zhang

**Affiliations:** 1 Department of Oncology, The Central Hospital of Wuhan, Tongji Medical College, Huazhong University of Science and Technology, Wuhan, Hubei, China; 2 Department of Radiation Oncology, State Key Laboratory of Oncology in South China, Guangdong Provincial Clinical Research Center for Cancer, Sun Yat-sen University Cancer Center, Guangzhou, Guangdong, China

**Keywords:** failed or deficient anti *Helicobacter pylori* treatment, gastric MALT lymphoma, radiotherapy, retrospective study, survival prognosis

## Abstract

**Objective:**

To investigate the impact of different first-line anti-tumor therapies on survival outcomes in patients with stage I-II gastric MALT lymphoma who failed or did not receive anti-*Helicobacter pylori* (*H. pylori*) therapy.

**Methods:**

Clinical data from 144 patients with anti-*H. pylori* therapy-failed or -naïve stage I-II gastric MALT lymphoma treated at Sun Yat-sen University Cancer Center between June 1998 and July 2021 were retrospectively analyzed. Patients were divided into three groups based on first-line treatment: systemic therapy alone radiotherapy alone, and radiotherapy combined with systemic therapy. Kaplan-Meier analysis and log-rank tests were used to compare progression-free survival (PFS) and overall survival (OS) among groups. Univariate and multivariate Cox regression analyses were performed to identify independent prognostic factors.

**Results:**

Significant differences were observed among the three groups regarding staging, H. pylori status, prior anti-H. pylori therapy, elevated LDH, Ki67%, and CD20+ expression (p < 0.05). With a median follow-up of 59 months (IQR: 35–101 months), the radiotherapy-alone group (n = 56) demonstrated superior PFS (p = 0.001), and OS (p = 0.031) compared to the systemic therapy-alone group (n = 39). No significant differences in PFS (p = 0.358) or OS (p = 0.386) were observed between the radiotherapy-alone and combined therapy groups (n= 49). Stratification by radiotherapy dose (≤30 Gy, 31–35 Gy, 36–42 Gy) revealed no survival differences (p > 0.05). Univariate analysis identified low LDH, low MALT-IPI score, prior anti-H. pylori therapy, and radiotherapy (alone or combined) as protective factors against recurrence (p < 0.05). However, multivariate analysis confirmed only radiotherapy (alone or combined) as independent predictors of reduced recurrence (p < 0.05).

**Conclusion:**

Radiotherapy remains the highly effective option for anti-H. pylori therapy-failed or -naïve stage I-II gastric MALT lymphoma. Combined therapy provided no additional survival benefit over radiotherapy alone. Notably, prior (even failed) anti-H. pylori therapy may reduce recurrence risk, warranting further validation.

## 1 Introduction

Gastric mucosa-associated lymphoid tissue (MALT) lymphoma is characterized by low incidence and prolonged disease course (1). It predominantly affects middle-aged and elderly individuals, with a higher prevalence in males than females, accounting for 1%–5% of all malignant tumor patients (2). Clinical manifestations vary significantly among patients after onset, ranging from asymptomatic cases in mild presentations to symptoms such as abdominal pain, dyspepsia, acid reflux, bloating, melena, and nausea in severe cases, though these symptoms lack specificity (3, 4).

Despite its low incidence, the number of patients has shown a marked upward trend in recent years. Studies (5, 6) indicate that Helicobacter pylori (H. pylori) infection is the primary cause of gastric MALT lymphoma development and progression. However, not all cases are H. pylori-related, with 70%–90% of patients showing concurrent H. pylori infection in clinical practice (7, 8). For H. pylori-positive patients, eradication therapy is considered the first-line treatment, achieving an overall response rate of 83% (9). Additional therapeutic options include chemotherapy (10), radiotherapy (11, 12), surgery (13), and immunotherapy (14).

Although most patients achieve complete remission after anti-H. pylori therapy, some fail to eradicate the infection and require further treatment (15). Current clinical guidelines recommend anti-H. pylori therapy and radiotherapy as first-line treatments for early-stage gastric MALT lymphoma (16, 17). However, real-world practice reveals diverse treatment approaches for early-stage patients, who are anti-H. pylori therapy-failed or -naïve, including systemic therapies such as chemotherapy or immunotherapy. For patients with stage I-II gastric MALT lymphoma who experience anti-H. pylori treatment failure or lack initial eradication, how should treatment strategies be optimized?

This study retrospectively analyzed 144 cases of stage I-II gastric MALT lymphoma patients with failed or absent anti-H. pylori treatment at our institution, aims to investigate the impact of different antitumor therapies on survival outcomes.

## 2 Methods

### 2.1 Patient eligibility

**Figure 1:**
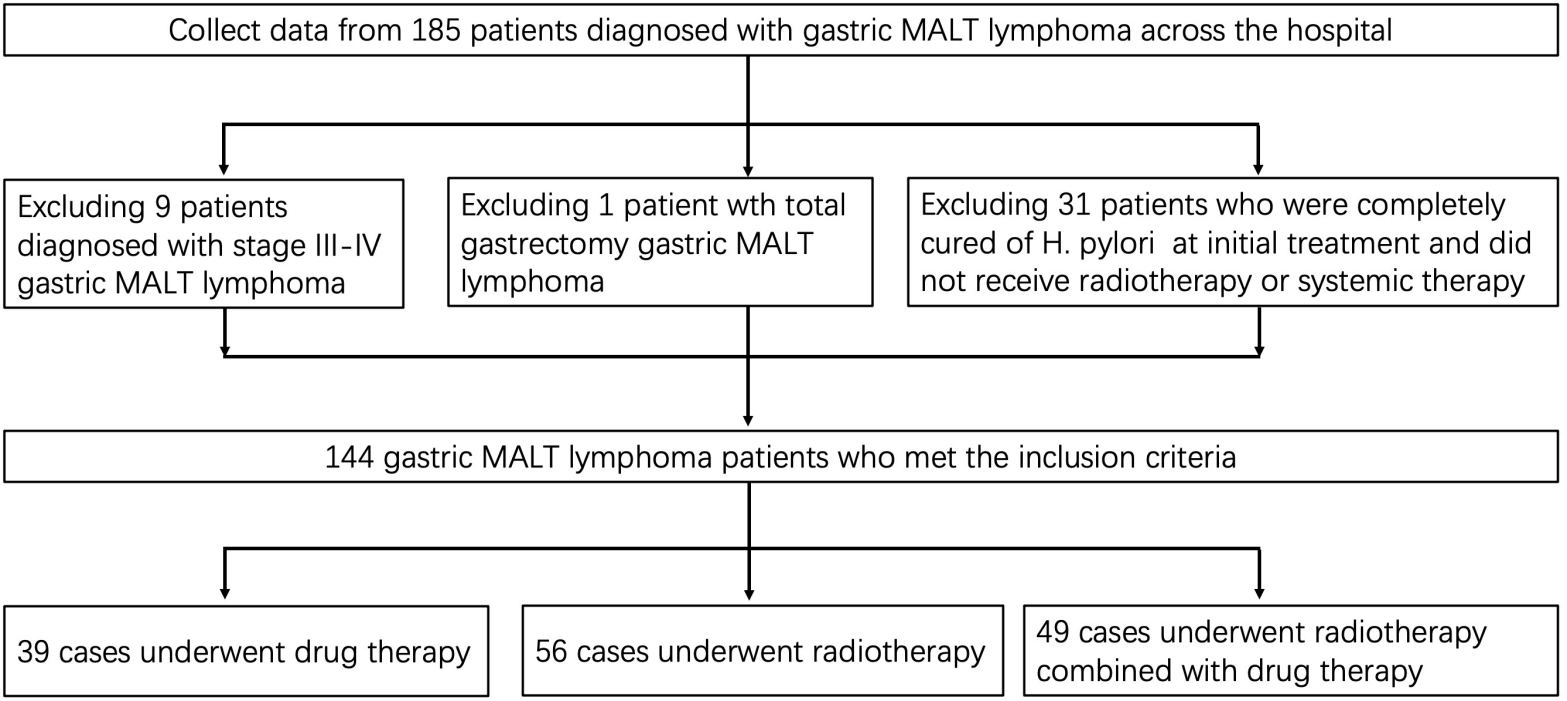
The research flow chart of patient screening.

Patients with gastric MALT lymphoma treated at Sun Yat-sen University Cancer Center between June 1998 and July 2021 were retrospectively reviewed (Ethics approval number: B2022-596-01). Inclusion criteria were: (1) primary lesion located in the stomach; (2) pathologically confirmed gastric MALT lymphoma; (3) Ann Arbor stage I-II gastrointestinal lymphoma; (4) complete clinical and imaging data; (5) post-treatment follow-up duration ≥6 months. Exclusion criteria included: (1) non-primary gastric MALT pathology; (2) Ann Arbor stage III-IV lymphoma; (3) complete remission after H. pylori eradication therapy alone; (4) patients who underwent total gastrectomy; (5) no radiotherapy or systemic therapy (including chemotherapy, targeted therapy, or immunotherapy); (6) follow-up duration <6 months; (7) concurrent other malignancies. Staging was based on the Ann Arbor staging system, determined via CT, PET-CT, and gastroscopy. Failed anti-H. pylori therapy is defined as the absence of histological or endoscopic improvement after at least one standard course of eradication therapy. The flowchart for screening patients is detailed in **Figure 1**.

### 2.2 Radiotherapy protocol

Radiotherapy was delivered using 3D conformal radiotherapy (3D-CRT) or intensity-modulated radiotherapy (IMRT). The clinical target volume (CTV) included the entire stomach and regional lymph nodes (perigastric, celiac, porta hepatis). The planning target volume (PTV) was defined as CTV + 1–1.5 cm margin. Fractionation schemes included:30 Gy,30~35 Gy, and 36–42 Gy.

### 2.3 Statistical analysis

PFS was defined as the time from diagnosis to disease progression or death from any cause. OS was defined as the time from diagnosis to death from any cause, last follow-up, or the cutoff observation date. Continuous variables were compared using the t-test. Categorical variables were analyzed using the chi-square test or Fisher’s exact test. Survival rates were calculated using the Kaplan-Meier method, and between-group comparisons were performed with the log-rank test. Univariate analysis and multivariate Cox proportional hazards regression models were used to evaluate independent prognostic factors. All analyses were two-tailed, with p <0.05 considered statistically significant. Statistical analyses were conducted using SPSS 26.0 software and R software (version 4.2.2).

## 3 Results

### 3.1 General clinical characteristics of the overall cohort

Among the 144 gastric MALT lymphoma patients, 90 were male and 54 were female, with a mean age at diagnosis of 53 years. Patients were classified as stage I (61.8%) or stage II (38.2%) based on Ann Arbor staging. The MALT-IPI score was predominantly 0 (84.7%) or 1 (15.3%), and elevated lactate dehydrogenase (LDH) levels were observed in 5.6% of patients. Endoscopic findings revealed the most common site of involvement as the gastric body (61.1%), followed by the gastric antrum (29.2%), with the gastric fundus being less frequently affected (6.1%). A Ki67 index <10% was observed in 68.8% of patients. Chromosomal translocation testing, including t (11, 18)(q21;q21) *MALT-API* and t (14, 18)(q32;q21) *MALT-IGH*, was performed in 50% of the cohort, with 25% of tested patients showing positive results (**Table 1**).

**Table 1:** Patients characteristics.

Characteristic		N (%)
All patients		144
Sex		
	Female	54 (37.5)
	Male	90 (62.5)
Age (years)		
	Median(range)	53.(45-81)
Year of diagnosis		
	1998-2007	17 (11.8)
	2008-2017	67 (46.5)
	2018-2021	60 (41.7)
Ann Arbor stage		
	I	89 (61.8)
	II	55 (38.2)
HP status		
	Negative	28 (19.4)
	Positive	96 (66.7)
	Unknow	20 (13.9)
Anti-HP therapy		
	No	50 (34.7)
	Yes	94 (65.3)
MALT-IPI		
	0	122 (84.7)
	1	22 (15.3)
LDH > UNL		
	No	136 (94.4)
	Yes	8 ( 5.6)
Primary lesion		
	Multiple	46 (31.9)
	Single	98 (68.1)
Ki67%		
	≤10	99 (68.8)
	11~60	20 (13.9)
	Unknow	25 (17.4)
Ectopic chromosome		
	No	36 (25.0)
	Unknow	72 (50.0)
	Yes	36 (25.0)
CD20+		
	Negative	18 (12.5)
	Positive	84 (58.3)
	Unknow	42 (29.2)
R		
	No	81 (56.2)
	Yes	63 (43.8)
Radiotherapy		
	No	39 (27.1)
	Yes	105 (72.9)
Radiotherapy dose		
	30-35Gy	83 (57.6)
	30Gy	6 ( 4.2)
	36-42Gy	14 ( 9.7)
	No	41 (28.5)
Chemotherapy		
	No	74 (51.4)
	Yes	70 (48.6)
Surgery		
	No	132 (91.7)
	Yes	12 ( 8.3)
First line treatment		
	Pharmacotherapy	39 (27.1)
	Radiotherapy	56 (38.9)
	RT+PT	49 (34.0)

MALT-IPI, MALT lymphoma prognostic index; LDH, Lactate dehydrogenase; UNL, Upper normal limit; R, Rituximab; RT+PT, Radiotherapy and Pharmacotherapy.

### 3.2 Recurrence and survival outcomes in the overall cohort

**Figure 2:**
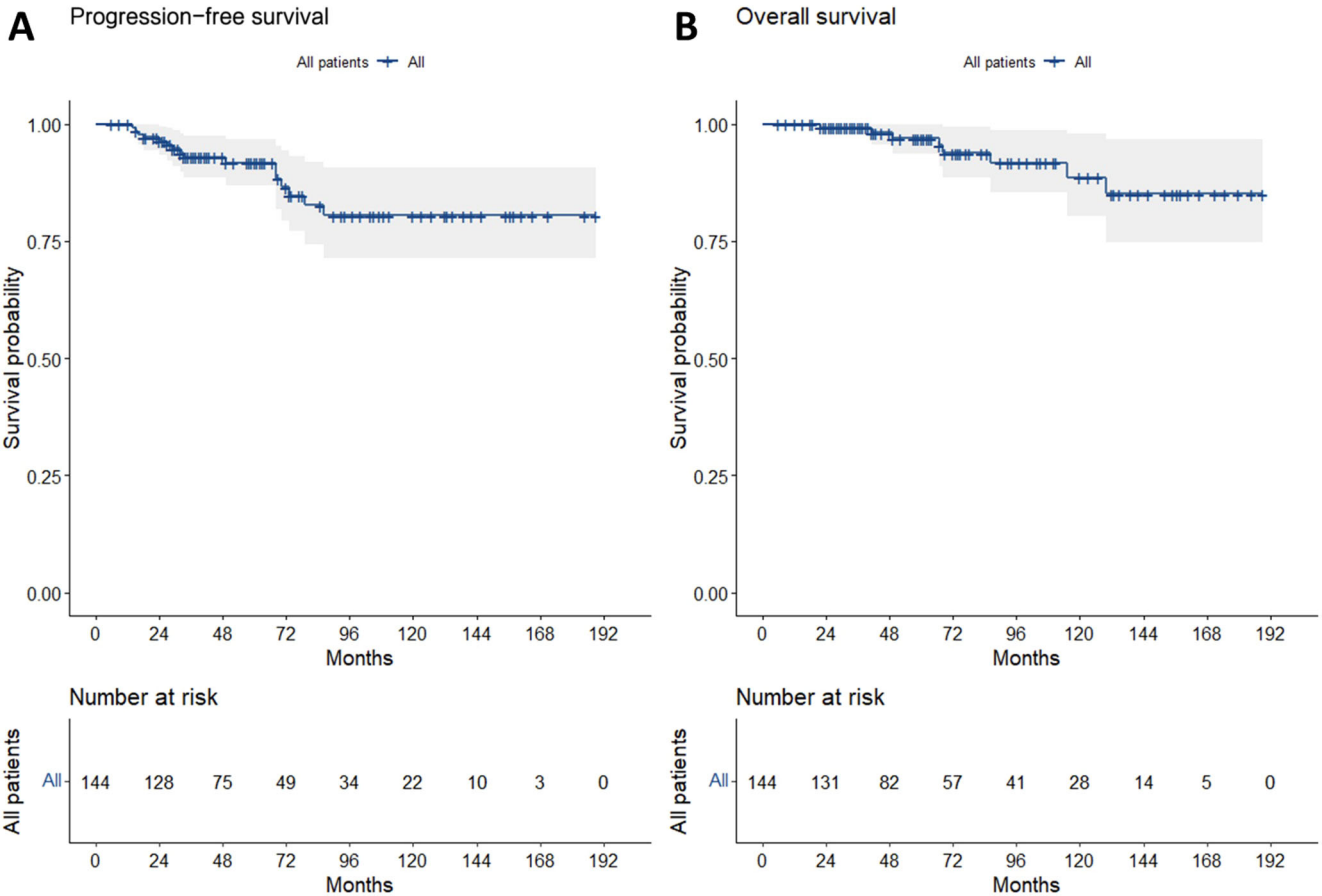
Long-term survival outcomes and disease progression patterns. PFS **(A)** and OS **(B)**.

The last follow-up date was December 1, 2022, with a median follow-up duration of 59 months (interquartile range: 34–103 months) for the entire cohort. The 5-year, 10-year, and 15-year PFS rates were 91.7%, 80.5%, and 80.5%, respectively, while the OS rates were 97.0%, 88.6%, and 85.1% at the same intervals (**Figure 2**). Tumor progression was documented in 14 patients. At initial progression, 8 patients experienced gastric recurrence, and 6 developed distant metastases. Among patients with localized recurrence, salvage therapies included systemic therapy (2 cases), salvage radiotherapy (2 cases), combined radiotherapy, and systemic therapy (2 cases), while 2 patients declined further treatment. Four patients developed distant metastases following localized progression. One case exhibited histological transformation to diffuse large B-cell lymphoma during progression.

### 3.3 Comparison of clinical characteristics and survival outcomes across three treatment groups

Patients were stratified into three groups based on first-line antitumor treatment: systemic therapy group (n=39), radiotherapy-alone group (n=56), and radiotherapy combined with systemic therapy group (n=49). General clinical characteristics across the groups are summarized in **Table 2**.

**Table 2:** Baseline characteristics of 144 patients with early-stage gastric MALT lymphoma, stratified by First line treatment.

Characteristic	Pharmacotherapy n=39(%)	Radiotherapy n=56(%)	RT+PT n=49(%)	*p*-Value
Sex				
Female	14 (35.9)	21 (37.5)	19 (38.8)	0.962
Male	25 (64.1)	35 (62.5)	30 (61.2)	
Age (years)				
mean (SD)	53 (12.3)	54 (12.5)	51(14.1)	0.49
Year of diagnosis				
1998-2007	14 (35.9)	0 (0.0)	3 (6.1)	<0.001*
2008-2017	15 (38.5)	22 (39.3)	30 (61.2)	
2018-2021	10 (25.6)	34 (60.7)	16 (32.7)	
Ann Arbor stage				
I	21 (53.8)	43 (76.8)	25 (51.0)	0.012
II	18 (46.2)	13 (23.2)	24 (49.0)	
HP status				
Negative	6 (15.4)	14 (25.0)	8 (16.3)	<0.001
Positive	16 (41.0)	41 (73.2)	39 (79.6)	
Unknow	17 (43.6)	1 (1.8)	2 (4.1)	
Anti-HP therapy				
No	27 (69.2)	13 (23.2)	10 (20.4)	<0.001
Yes	12 (30.8)	43 (76.8)	39 (79.6)	
MALT-IPI				
0	29 (74.4)	49 (87.5)	44 (89.8)	0.103
1	10 (25.6)	7 (12.5)	5 (10.2)	
LDH > UNL				
No	33 (84.6)	55 (98.2)	48 (98.0)	0.008*
Yes	6 (15.4)	1 (1.8)	1 (2.0)	
Primary lesion				
Multiple	17 (43.6)	11 (19.6)	18 (36.7)	0.033
Single	22 (56.4)	45 (80.4)	31 (63.3)	
Ki67%				
≤10	19 (48.7)	43 (76.8)	37 (75.5)	<0.001
11~60	4 (10.3)	7 (12.5)	9 (18.4)	
Unknow	16 (41.0)	6 (10.7)	3 (6.1)	
Ectopic chromosome				
No	5 (12.8)	19 (33.9)	12 (24.5)	0.071
Unknow	26 (66.7)	21 (37.5)	25 (51.0)	
Yes	8 (20.5)	16 (28.6)	12 (24.5)	
CD20+				
Negative	9 (23.1)	4 (7.1)	5 (10.2)	0.028
Positive	17 (43.6)	32 (57.1)	35 (71.4)	
Unknow	13 (33.3)	20 (35.7)	9 (18.4)	
Radiotherapy dose				
30-35Gy	0 (0.0)	49 (87.5)	34 (69.4)	<0.001
30Gy	0 (0.0)	1 (1.8)	5 (10.2)	
36-42Gy	0 (0.0)	4 (7.1)	10 (20.4)	
No	39 (100.0)	2 (3.6)	0 (0.0)	
Disease progression				
Distant	8 (20.5)	1 (1.8)	0 (0.0)	<0.001*
Local	5 (12.8)	3 (5.4)	1 (2.0)	
No	26 (66.7)	52 (92.9)	48 (98.0)	
Final outcome				
Alive	32 (82.1)	56 (100.0)	48 (98.0)	<0.001*
Death	7 (17.9)	0 (0.0)	1 (2.0)	
Overall survival(months)				
Median	76	40	62	0.015
IQR	35-133	24-73	40-97	
*Fisher's exact test;MALT-IPI = MALT lymphoma prognostic index;					

LDH, Lactate dehydrogenase; UNL, Upper normal limit; LNL, Lower normal limit;

Significant differences (p<0.05) were observed among the groups in terms of disease stage, H. pylori infection status, anti-H. pylori treatment, elevated LDH levels, Ki67 index, and CD20 positivity. For stage I patients, the proportion treated with radiotherapy alone was higher compared to other groups. Among H. pylori-positive patients, approximately 80% in the radiotherapy-alone and combined therapy groups received standard anti-H. pylori treatment. In contrast, patients with unknown H. pylori status were more likely to receive systemic therapy, with most initial treatments occurring before 2007.The 5-, 10-, and 15-year PFS rates were 73.9%, 56.5%, and 56.5% in the systemic therapy group; 100%, 84%, and not reached in the radiotherapy-alone group; and 96.9%, 96.9%, and 96.9% in the combined therapy group (p<0.0001). Corresponding OS rates were 93.7%, 75.9%, and 70.0% (systemic therapy); 100%, 100%, and not reached (radiotherapy-alone); and 96.9%, 96.9%, and 96.9%(combined therapy) (p=0.014). The radiotherapy-alone group showed superior PFS (p=0.001) and OS (p=0.031) compared to the systemic therapy group, while no significant differences were observed between the combined therapy and radiotherapy-alone groups in PFS (p=0.358) or OS (p=0.386) (**Figure 3**). Despite multivariate adjustment, residual confounding may persist due to baseline imbalances in stage, H. pylori status, and biomarker expression between groups.

**Figure 3:**
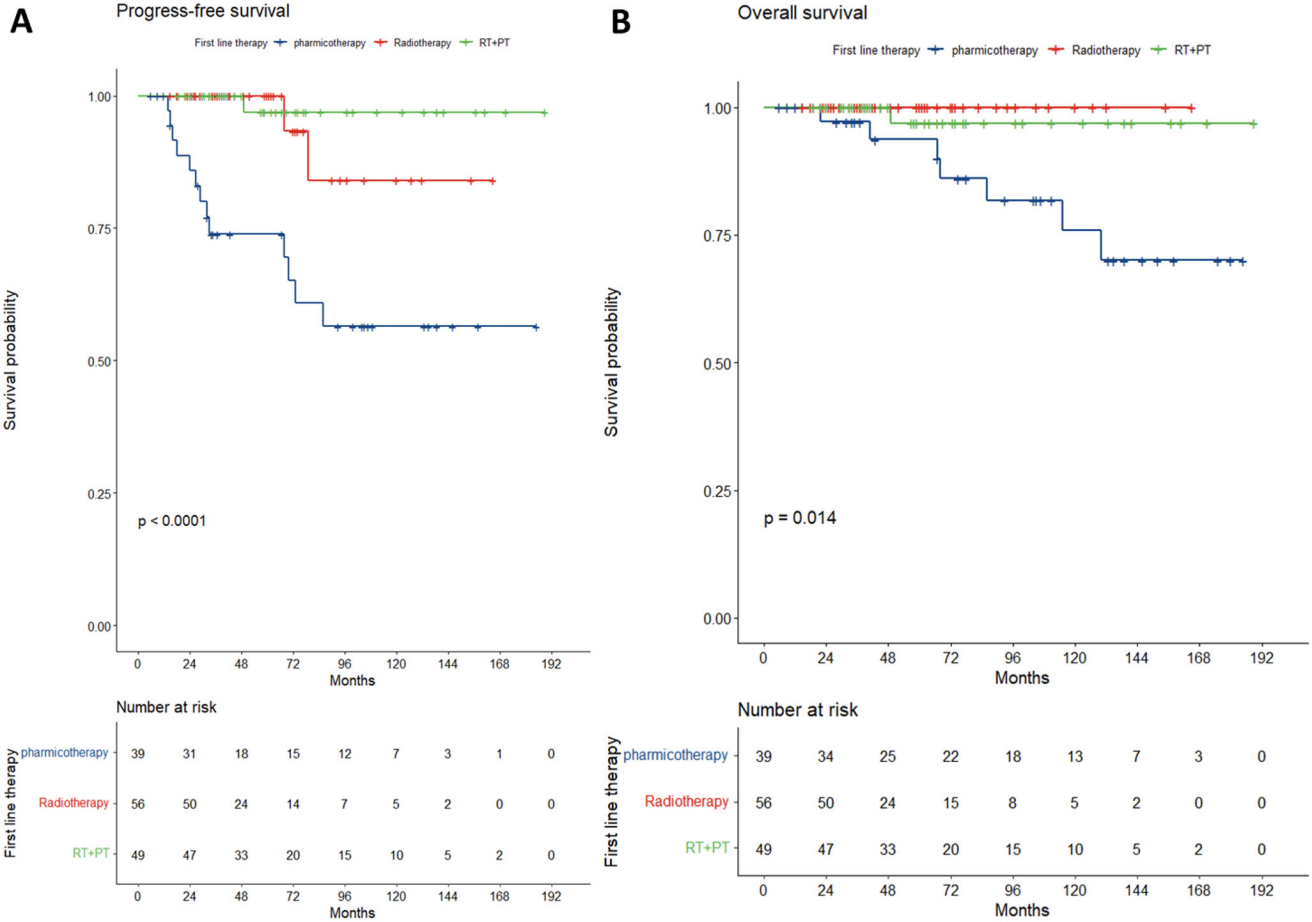
Kaplan-meier survival curves by treatment group. **(A)** (PFS) and **B** (OS).

### 3.4 Impact of radiotherapy dose on survival

**Figure 4:**
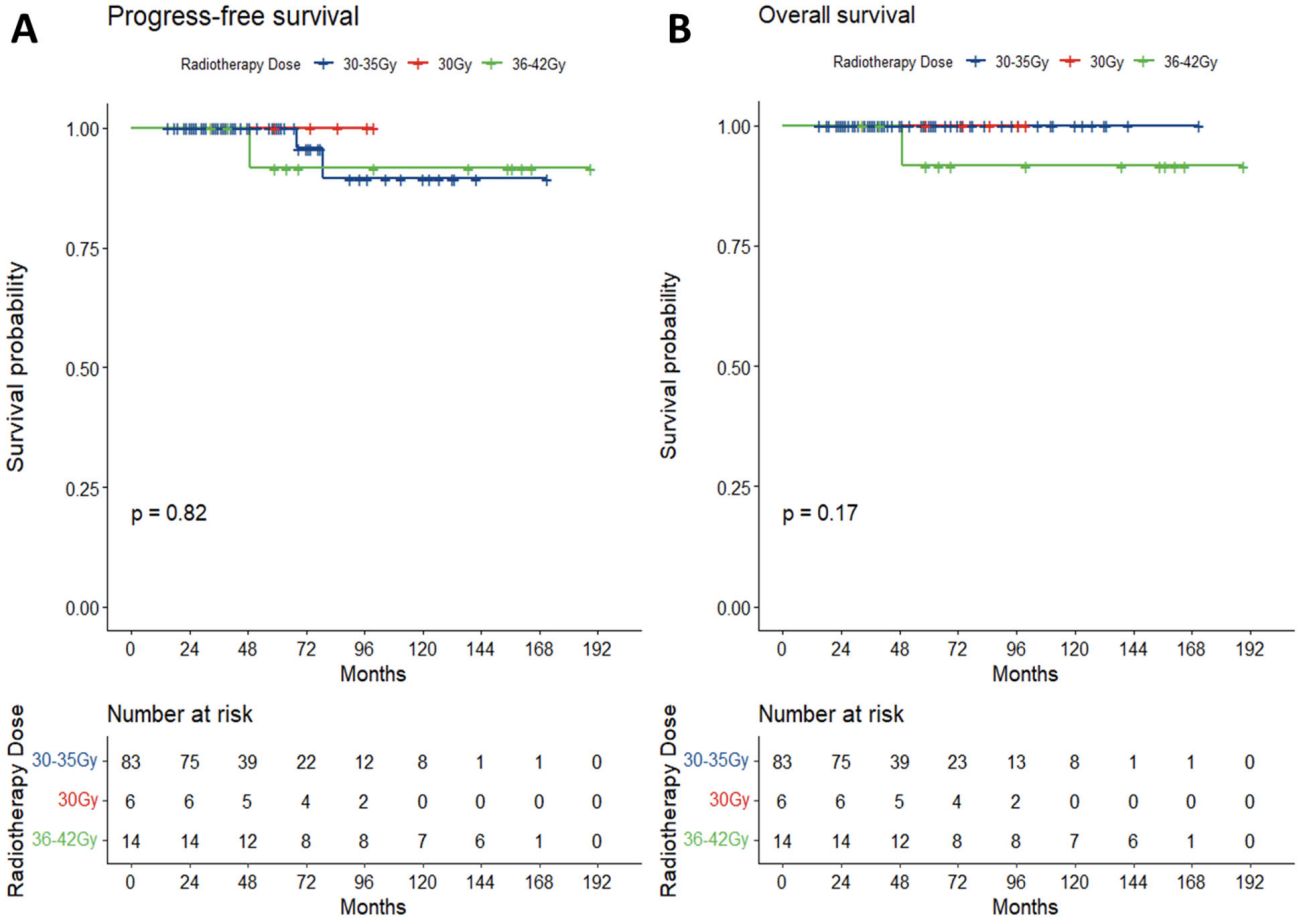
Radiotherapy dose subgroup analysis. PFS **(A)** and OS **(B)** by radiotherapy dose group. Numbers at risk at 5, 10, and 15 years are indicated below each panel.

Patients receiving radiotherapy were divided into three dose groups: ≤30 Gy, 31–35 Gy, and 36–42 Gy. Log-rank analysis revealed no statistically significant differences in PFS (p=0.82) or OS (p=0.17) among the groups (**Figure 4**).

### 3.5 Prognostic factors for recurrence and metastasis in early-stage gastric MALT lymphoma

Univariate and multivariate Cox regression analyses identified elevated LDH (HR: 5.28, 95% CI: 1.16–16.57), higher MALT-IPI score (HR: 3.16, 95% CI: 1.14–8.72), anti-H. pylori treatment (HR: 0.3, 95% CI: 0.11–0.82), and first-line antitumor treatment strategy as significant prognostic factors for PFS (p<0.05). Elevated LDH and higher MALT-IPI scores were risk factors for disease progression, while prior anti-H. pylori therapy, radiotherapy alone (HR: 0.12, 95% CI: 0.03–0.55), and combined radiotherapy-systemic therapy (HR: 0.05, 95% CI: 0.01–0.41) were protective factors. However, in multivariate analysis, only first-line antitumor treatment remained an independent prognostic factor for PFS, likely due to the limited sample size. These findings require validation through multicenter studies with larger cohorts (**Table 3**).

**Table 3:** Univariate and multivariate Cox regression analyses for progression-free survival of the early-stage gastric MALT lymphoma patients.

Characteristic	Univariate		Multivariate	
	HR(95%CI)	*P*	HR(95%CI)	*P*
Age	1.03 (0.99-1.07)	0.11	1.05(0.99-1.12)	0.0902
Anti-HP therapy				
No	Reference		Reference	
Yes	0.3 (0.11-0.82)	0.02	0.91(0.23-3.62)	0.8915
Ectopic chromosome				
No	Reference		Reference	
Unknow	4.47 (0.57-34.94)	0.154	3.65(0.14-32.38)	0.2451
Yes	5.68 (0.66-48.69)	0.113	8.25(0.83-82.04)	0.0719
LDH > UNL				
No	Reference		Reference	
Yes	5.28 (1.68-16.57)	0.004	5.57(0.62-50.29)	0.1262
MALT IPI				
0	Reference		Reference	
1	3.16 (1.14-8.72)	0.027	0.32(0.04-2.51)	0.2768
Ann Arbor stage				
I	Reference		Reference	
II	1.79 (0.67-4.78)	0.243	2.56(0.82-7.98)	0.1052
First line treatment				
Pharmacotherapy	Reference		Reference	
Radiotherapy	0.12 (0.03-0.55)	0.006	0.12(0.02-0.67)	0.0152
RT+PT	0.05 (0.01-0.41)	0.005	0.05(0.01-0.46)	0.0077

## 4 Discussion

This retrospective analysis of 144 patients with stage I-II gastric MALT lymphoma who experienced failed or lacked anti-*Helicobacter pylori* (H. pylori) therapy highlights the central role of radiotherapy in improving survival outcomes for this population. The results demonstrated significantly superior PFS and OS in the radiotherapy-alone group compared to the systemic therapy group, while combined radiotherapy and systemic therapy did not further enhance survival. Additionally, prior anti-*H. pylori* treatment was associated with reduced recurrence risk, offering novel insights for clinical practice.

This study confirms radiotherapy as an effective treatment for stage I-II gastric MALT lymphoma patients with failed or absent anti-*H. pylori* therapy. The radiotherapy-alone group achieved 5-year PFS and OS rates of 100% and 100%, respectively, significantly outperforming the systemic therapy group (73.9% and 93.7%). These findings align with NCCN guidelines recommending radiotherapy as the preferred option for early-stage gastric MALT lymphoma. Notably, combining radiotherapy with systemic therapies (e.g., chemotherapy or immunotherapy) did not improve survival, suggesting limited benefit from systemic treatments in localized disease. Furthermore, no survival differences were observed across radiotherapy dose groups (≤30 Gy, 31–35 Gy, 36–42 Gy), indicating that lower doses (e.g., 30 Gy) may suffice for local control, consistent with recent trends advocating dose de-escalation.

While radiotherapy alone demonstrated superior survival outcomes, it was associated with minimal toxicity (primarily Grade 1–2 nausea/fatigue). In contrast, systemic medication (e.g., chemotherapy/rituximab) increased hematologic and constitutional toxicity. Combined therapy exacerbated these effects without survival benefit, reinforcing radiotherapy alone as the optimal balance of efficacy and safety for localized disease.

While prior studies emphasize the pivotal role of *H. pylori* eradication in gastric MALT lymphoma, optimal strategies for treatment-refractory or *H. pylori*-negative patients remain debated (18–20). This study revealed that even after failed anti-*H. pylori* therapy, patients who received such treatment exhibited reduced recurrence risk (HR: 0.3, 95% CI: 0.11–0.82), suggesting that eradication may indirectly suppress tumor progression via microenvironment modulation or immune response. This observation resonates with hypotheses proposing anti-*H. pylori* therapy for *H. pylori*-negative patients, though mechanistic validation is warranted.

The superiority of radiotherapy in our cohort aligns with findings from the prospective study by Laurent et al. (21). However, the lack of additional benefit from combined therapy contrasts with small-scale studies reporting improved outcomes with radiotherapy plus rituximab, possibly due to population heterogeneity or regimen differences.

Our study is limited by its retrospective design and significant differences in baseline characteristics among treatment groups (**Table 2**). Although multivariate Cox regression was performed, unmeasured confounders (e.g., physician preference for therapy selection, undocumented comorbidities) may influence outcomes. For instance, earlier-stage patients were more likely to receive radiotherapy alone, potentially amplifying its observed benefit. These biases underscore the need for prospective validation.

This study provides critical evidence supporting radiotherapy as the first-line treatment for stage I-II gastric MALT lymphoma patients with failed or absent anti-H. pylori therapy, while urging cautious evaluation of combined therapies. Future high-quality studies are needed to refine therapeutic strategies and achieve personalized treatment goals.

## Data Availability

The raw data supporting the conclusions of this article will be made available by the authors, without undue reservation.
